# Novel Immunomodulatory Proteins Generated via Directed Evolution of Variant IgSF Domains

**DOI:** 10.3389/fimmu.2019.03086

**Published:** 2020-01-21

**Authors:** Steven D. Levin, Lawrence S. Evans, Susan Bort, Erika Rickel, Katherine E. Lewis, Rebecca P. Wu, Joseph Hoover, Sean MacNeil, David La, Martin F. Wolfson, Mark W. Rixon, Stacey R. Dillon, Michael G. Kornacker, Ryan Swanson, Stanford L. Peng

**Affiliations:** ^1^Alpine Immune Sciences Inc., Seattle, WA, United States; ^2^Department of Biochemistry, University of Washington, Seattle, WA, United States

**Keywords:** costimulation, protein engineering, protein therapeutics, anti-inflammatory, IgSF, ICOS ligand (ICOSL)

## Abstract

Immunoglobulin superfamily member (IgSF) proteins play a significant role in regulating immune responses with surface expression on all immune cell subsets, making the IgSF an attractive family of proteins for therapeutic targeting in human diseases. We have developed a directed evolution platform capable of engineering IgSF domains to increase affinities for cognate ligands and/or introduce binding to non-cognate ligands. Using this scientific platform, ICOSL domains have been derived with enhanced binding to ICOS and with additional high-affinity binding to the non-cognate receptor, CD28. Fc-fusion proteins containing these engineered ICOSL domains significantly attenuate T cell activation *in vitro* and *in vivo* and can inhibit development of inflammatory diseases in mouse models. We also present evidence that engineered ICOSL domains can be formatted to selectively provide costimulatory signals to augment T cell responses. Our scientific platform thus provides a system for developing therapeutic protein candidates with selective biological impact for treatments of a wide array of human disorders including cancer and autoimmune/inflammatory diseases.

## Introduction

Immune cells communicate and coordinate responses to pathogens through a complex series of protein-protein interactions. Many of the molecules sensing external signals on immune cells are members of the immunoglobulin super family (IgSF) (1) and considerable effort has been put into understanding how they function and how they might be manipulated when immune responses are inadequate or overly robust. Inadequate responses can lead to a failure to control viral infection or tumor development/growth, while overly robust responses can trigger collateral damage to tissues and organs resulting in clinical manifestations of autoimmune diseases. Importantly, IgSF proteins can contribute to activation (2) as well as inhibition of immune cells (3), and a number of therapeutic strategies targeting IgSF members have been employed to appropriately regulate immune responses.

T cells are activated primarily through engagement of the T cell antigen receptor (TCR), but sustained T cell activation also requires signaling from what have been termed costimulatory receptors. Two of the more important costimulatory receptors are the IgSF proteins CD28 and Inducible Costimulator (ICOS). CD28 signals are triggered through engagement by the cognate ligands CD80 and CD86, whereas ICOS-mediated costimulation is driven by Inducible Costimulator Ligand (ICOSL). Absence or blockade of these signals results in poor or incomplete T cell activation, which can be advantageous in tempering immune responses in autoimmune or inflammatory conditions, but deleterious for immune responses to tumors. For the treatment of malignant disease, many recent therapies have focused on augmentation of responses, including increasing costimulatory signals at the tumor site.

Protein therapeutics have been widely used to target key members of the IgSF to effectively manipulate immune responses. Antibodies have been directed against inhibitory receptors or their counter-structures to augment the response against tumors by releasing cells from inhibitory signals (4), while antibodies and soluble forms of IgSF receptors have been developed to block important immuno-modulatory pathways to temper destructive immune responses in autoimmunity (5) or to facilitate immune cell activation in malignant disease. However, soluble forms of IgSF proteins generally have low affinities for their native counter-structures (6), which can limit their efficacy.

To circumvent this shortcoming, we have developed a platform using yeast display and directed evolution of IgSF proteins to produce variant immunoglobulin domains (termed “vIgDs”) with increased affinity for their natural counter-structures. Importantly, the strategy relies on the introduction of small numbers of random mutations in individual clones within a large library of coding sequences. The resulting proteins are then subjected to binding-based selection for higher affinity clones. Further, we've found that in addition to engineering higher affinities for cognate ligands, we can simultaneously select for variants that also bind counter-structures not normally, or minimally bound by the native protein. We present here the results of such efforts using ICOSL, where our scientific platform has been used to generate variants with enhanced binding to the native T cell costimulatory counter-structure ICOS. In addition, we were able to engineer variant single molecules of ICOSL with high affinity for both CD28 and ICOS. Moreover, we show that depending on choice of molecular format of these variant ICOSL molecules, their function can be altered to either block or deliver costimulatory signals through the targeted receptors, CD28 and ICOS. Hence, therapeutic candidate proteins containing the engineered ICOSL variant domains described here have the potential to moderate T cell activation or accentuate T cell responses, thus allowing generation of therapeutic proteins useful in treatment of autoimmune/inflammatory diseases and cancer.

## Methods

### Yeast Library Selections

Random libraries including the coding sequence for the extracellular domain (ECD) of wild-type human ICOSL were generated by error-prone PCR (GeneMorph, Agilent). The reaction was adjusted to generate an average of three to five codon mutations per variant resulting in amino acid substitutions. Mutated DNA was expanded using PCR with primers adding 30 bp overhangs for homologous recombination of PCR product with yeast display vector (Life Technologies) after electroporation into yeast. The final protein displayed on yeast contained an N-terminal HA tag followed by ICOSL ECD followed by the yeast cell surface protein SAG1P. Libraries were introduced into yeast by electroporation essentially as described (7). Library size was ~10^8^. For library selection, 10^9^ transformants were grown in non-inducing selective medium, followed by sub-culturing 10^9^ cells for 2 days at 30°C in the same medium with galactose to induce expression of display fusion proteins on the yeast cell surface. To generate a population of cells enriched for ICOS and/or CD28 binding, cells were processed by bead sorting using Protein A-coated magnetic beads (New England Biolabs) coated with CD28-Fc or ICOS-Fc (R&D Systems) essentially as described (7). This generally resulted in an output of up to 10^6^ variants which were then grown with 10-fold oversampling for the first round of fluorescence-activated cell sorting (FACS) using the same proteins used for bead sorting. Subsequent FACS were always carried out with a 10-fold oversampling of the outputs from prior sorts. For second generation library generation and sorting, plasmid was isolated from pooled first-generation selection outputs (Zymoprep, Zymoresearch) and then used as DNA template for a new GeneMorph reaction, library generation, and sorting. To identify mutations in variants from yeast display, the ICOSL DNA inserts from yeast outputs were batch cloned into an Fc-fusion expression vector and up to 100 individual transformants were then DNA sequenced to identify unique variants.

### Expression Vectors, Transfections, and Lentiviral Infections

For production of proteins, engineered domains identified by yeast display were cloned individually or as fusions with other engineered domains into a derivative of vector pcDNA3.4 (Thermofisher) containing an IgG1 Fc domain lacking effector function. Inserts were positioned downstream of the CMV promoter and upstream of the Fc module to generate Fc-fusions. Inserts were preceded by a Kozak sequence immediately followed by the human immunoglobulin VH5-51 signal peptide to direct secretion of constructs into culture medium. Open reading frames were terminated with stop codons TAA or TGA. Gly_4_Ser linkers were introduced between fused domains.

The ICOSL-NKp30 vIgD-Fc protein was generated by utilizing Gly_4_Ser linkers to fuse the ICOSL vIgD on the N-terminal side of the NKp30 vIgD domain which was then fused on the N-terminal side of the Fc domain.

Four different V-mAbs containing one of two ICOSL vIgD domains (A2239 or A2231, Supplementary Table 1) were generated by utilizing Gly_4_Ser linkers to fuse the ICOSL vIgD to either the N- or C-terminal end of either the heavy or light chain of trastuzumab (8). Four additional V-mAbs were generated containing four ICOSL vIgD domains by cotransfecting trastuzumab heavy and light chain constructs with each containing an ICOSL vIgD domain.

For cell surface expression of ICOSL and B7H6 on mammalian cells, DNA encoding full length proteins were cloned into the lentiviral vector pRRL downstream of an MND promoter and Kozak sequence located downstream of the cPPT element. All inserts were preceded by native signal peptides to direct expression at the cell surface. To allow for monitoring of lentiviral transduction efficiency and selection of stable integrants, protein expression was translationally coupled to EGFP or a puromycin resistance gene via an intervening T2A peptide (9). Lentiviral vectors, together with helper plasmids pMD2.G and psPAX2, were transfected into Expi293F™ cells to generate lentiviral particles. Viral particles were used to infect target cells for stable lentiviral expression (10). For some experiments, the pLVX-EF1α-IRES-Puro lentiviral vector, Lenti-X Packaging Single Shots packaging plasmids and Lenti-X 293 X packaging cell line were used to generate viral particles per the manufacturer's protocol (Clontech, Takara Bio). Viral supernatants were collected 48–72 h after transfection and in some cases concentrated using the Lenti-X^TM^ Concentration kit (Clontech, Takara Bio) per the manufacturer's protocol. Target cells were seeded in growth media in a 6-well plate (Corning) and 1 ml virus was added with Polybrene at a final concentration of 10 μg ml^−1^. Plates were spun at 30°C, at 2,500 RPM for 30 min and then incubated overnight at 37°C in 5% CO_2_. Media was changed the next day and expression of protein monitored by flow cytometry (FACS).

### Recombinant Protein Production and Purification

Recombinant vIgD Fc-fusion proteins and native IgSF receptor ECD Fc-fusion proteins were produced via transient expression in Expi293F™ cells obtained from Invitrogen Thermo Fisher Scientific following the manufacturer's recommended protocols. Protein was purified from the conditioned media harvests by capture and elution from Protein A (MabSelect SuRe, GE Healthcare) followed by an additional Size Exclusion Chromatography (SEC) polishing step (HiLoad 16/600 Superdex 200 pg, 1.6 × 60 cm, GE Healthcare) to generate monomeric, highly purified material as assessed by analytical SEC (Supplementary Table 3). Protein concentrations were determined according to the absorption at 280 nm using extinction coefficients calculated from the predicted primary structure (11). The purified proteins were formulated in 10 mM acetate, 9% sucrose, pH 5.0, vialed in a sterile biosafety cabinet and frozen at −80°C. A sample vial was thawed and assessed by analytical SEC to demonstrate stability after thaw. Analytical SEC was performed on a G3000SW XL column (7.8 mm × 30 cm, 5 μM, Tosoh Biosciences) at a flow rate of 0.5 μl min^−1^ using an Alliance 2695 (Waters) with 10 mM Acetate, 250 mM NaCl, pH 5 as running buffer. The purified proteins (50 μl) were applied at a concentration of 0.5 mg ml^−1^. Additionally, material from this vial was tested for endotoxin levels using the Charles River Laboratories LAL endotoxin kit. Endotoxin levels for all proteins were <1 EU mg^−1^.

### Determination of Binding Affinities by ForteBio Octet

Binding affinities between ICOSL vIgD-Fc and counter-structure receptors were determined on a Pall ForteBio Octet^®^ QK^e^ System. Soluble ICOS-Fc, CD28-Fc, or CTLA4-Fc-fusion proteins (R&D Systems) were individually loaded onto Protein A sensors, which were stripped and regenerated after each binding cycle using the standard ForteBio protocol. Wild type ICOSL-Fc, negative control wild type PDL2-Fc fusion, or the ICOSL vIgD-Fc were bound to the counter-structure receptors in four-point titrations. Each titration was globally fit to calculate the association (k_on_), dissociation (K_dis_), max response and dissociation constant (K_D_) of each protein. The K_D_ of the ICOSL vIgD-Fc were compared to wild type ICOSL-Fc to determine the fold difference in binding. As the engineered ICOSL vIgD-Fc and counter-structure receptors were both Fc dimers, an avidity component of the K_D_ measurements was assumed to be constant when comparing the K_D_-values.

### Cell-Based Binding Assays

Binding of ICOSL vIgD-Fc to ICOS, CD28, and CTLA4 was assessed on transiently transfected Expi293F cells. Briefly, Expi293F cells were transiently transfected with pcDNA3.1 expression vector including coding sequences for full length human CD28, CTLA4 or ICOSL (Genscript) using the ExpiFectamine 293 transfection kit (Life Technologies). Thirty micrograms of expression vector DNA in 1.5 ml ExpiFectamine was added to 75 million cells for 48 h prior to staining. Two hundred thousand transfected cells were incubated with ICOSL vIgD-Fc or control proteins over a range of concentrations from 100 nM to 100 pM in phosphate buffered saline (PBS; Gibco) supplemented with 0.5% bovine serum albumin, 5 mM EDTA and 0.1% sodium azide. Bound protein was detected with PE-conjugated anti-human-Fc (Jackson ImmunoResearch). FACS analysis was done on an LSRII (BD Biosciences) or a Hypercyt flow cytometer (Intellicyt). Data were analyzed using FlowJo software (Treestar) or Forcyte software (Intellicyt).

### Proposed ICOSL Protein Structure With Mutations

The structure for the ICOSL-ICOS complex was modeled by using the CD80-CTLA4 X-ray crystal structure (PDB ID 1I8L) as the template for homology modeling using the SWISS-MODEL server (12). The sequences of ICOSL and ICOS were aligned to CD80 and CTLA4 structures, respectively. Two models were generated independently: (1) the homo-dimer model for ICOSL, and (2) the bound hetero-complex between a single ICOSL unit and ICOS. Next, the full homology model was constructed by superimposing the ICOSL of the hetero-complex to each monomeric unit of the homo-dimer ICOSL model using PyMOL Molecular Graphics System. This produced the full ICOSL homo-dimer bound to ICOS, which was then subjected to a final round of energy minimization to produce the final homology model using Swiss-PDB Viewer.

### Mixed Lymphocyte Reactions (MLR)

Human primary dendritic cells (DC) were generated by culturing monocytes isolated from peripheral blood mononuclear cells (PBMC; Bloodworks Northwest) *in vitro* for 7 days with 50 ng ml^−1^ IL-4 and 80ng ml^−1^ GM-CSF (R&D Systems) in X-Vivo 15 media (Lonza). On days 3 and 5, half of the media was removed and replaced with fresh media containing cytokines. To fully induce DC maturation, lipopolysaccharide (LPS) (InvivoGen Corporation) was added at 100 ng ml^−1^ to the DC cultures on day 6 and cells were incubated for an additional 24 h. Ten thousand stimulated DC and 100,000 purified allogeneic human T cells (Bloodworks Northwest) were co-cultured with ICOSL vIgD-Fc or control proteins in 96-well round bottom plates in X-Vivo 15 media in a final volume of 200 μl. For some experiments, T cells were labeled with 0.25 μM CFSE (Invitrogen) for 10 min at room temperature prior to plating. Culture supernatants were collected on day 4 or 5 of culture and levels of IFNγ were analyzed using the Human IFNγ Duoset ELISA kit (R&D Systems). Optical density was measured on a BioTek Cytation Multimode Microplate Reader (BioTek Corporation) and quantitated against titrated recombinant IFNγ standard included in the IFNγ Duo-set kit. Cells were then stained for expression of cell surface markers using conjugated antibodies specific for human CD4 (RPA-T4), CD8 (RPA-T8), CD28 (CD28.2), and ICOS (C398.4A) (all from Biolegend). LIVE/DEAD Fixable Dead Cell stain (Life Sciences) was used to discriminate viable cells as directed by the manufacturer. Cells were then analyzed on an LSR II flow cytometer (BD Biosciences) for viability, expression of cell surface markers, and proliferation by CFSE dilution using the gating strategy outlined in Supplementary Figure 1.

### Plate Bound ICOSL vIgD-Fc Costimulation Assay

96-well flat bottom polystyrene tissue culture plates (Corning) were coated with a final concentration of 10, 3.3, 1.1, or 0.37 nM anti-CD3 (LEAF purified, clone OKT3; BioLegend) in the presence of 40 nM ICOSL vIgD-Fc or control proteins in PBS. Plates were incubated overnight at 4°C, then washed twice with PBS, and 100,000 either unlabeled T cells or, for some experiments, CFSE-labeled T cells in X-Vivo 15 media (Lonza) were added to each well. The cells were cultured in a 5% CO_2_ atmosphere at 37°C, and cells and supernatants were harvested at 72 h. Proliferation was measured via CFSE dilution and IFNγ ELISAs were run on the supernatants per the manufacturer's instructions (R&D systems).

### Plate Bound ICOSL-NKp30 vIgD-Fc Costimulation Assay

96-well flat bottom polystyrene tissue culture plates (Corning) were coated with a final concentration of 10 nM anti-CD3 antibody in the presence of varying concentrations of recombinant B7H6-Fc (R&D Systems) in PBS. Plates were incubated overnight at 4°C, then washed 2X with PBS. One hundred thousand T cells in X-Vivo 15 media were added to each well along with 40 nM of wild type ICOSL-Fc, wild type NKp30-Fc, ICOSL-NKp30 vIgD-Fc proteins, or control proteins in X-Vivo 15 media. The cells were cultured at 37°C, and cells and supernatants were harvested at 72 h. Proliferation and release of IFNγ were assessed as described above.

### K562-T Cell Co-culture Assays

K562 cells (ATCC) were either used untreated or, in some cases, were treated with 50 μg ml^−1^ mitomycin C (Life Technologies) per the manufacturer's instructions to arrest growth. For some experiments, K562 cells were labeled with CFSE to better distinguish them from T cells in co-culture assays. Purified primary human T cells were labeled with either CFSE or Cell Trace Far Red (both from Thermo-Fisher) and co-plated in a 96-well round bottom tissue culture plates with K562 cells, anti-CD3 antibody and the indicated ICOSL-NKp30 vIgD-Fc proteins, ICOSL vIgD-Fc, NKp30 vIgD-Fc or other control proteins. Cells were incubated 72 h and proliferation and IFNγ release were monitored as above.

### K562 Assays With Previously Stimulated T Cells

Purified T cells were incubated 2 days at 37°C with 100 ng/ml of plate-bound anti-CD3 antibody plus 1 ng/ml soluble anti-CD28 antibody. Stimulated T cells were phenotyped for CD28 and ICOS expression following stimulation and found to be 80–90% double positive for the two costimulatory receptors. These pre-stimulated T cells were then CFSE labeled and restimulated in the presence of K562 cells expressing a single-chain Fv fragment of OKT3 plus either ICOSL alone or a combination of ICOSL, CD80 and CD86. Proliferation was evaluated by CFSE dilution after 3 days in culture.

### Functional Assessment of Trastuzumab-ICOSL V-mAb Proteins

Expi293F cells (Life Technologies) were maintained in Expi293 expression medium at 37°C in a humidified atmosphere of 8% CO2 in air. CEM.T2, K562, and NCI-N87 (ATCC) cells were maintained in RPMI 1640 (Life Technologies) supplemented with 10% fetal bovine serum (FBS) (HyClone Laboratories), 1 mM sodium pyruvate, and antibiotics (penicillin 100 U ml^−1^ and streptomycin 100 μg ml^−1^) at 37°C, 5% CO2. Stable CEM.T2 cells expressing full-length human HER-2 and NCI-N87 cells expressing anti-CD3 single chain Fv (OKT3, NCI-N87-OKT3) were generated via spinfection with lentivirus encoding each protein and puromycin resistance markers. Transduced cells were selected with 1 μg ml^−1^ puromycin and were stained to confirm expression of desired surface protein.

Full-length mammalian surface expression constructs for human CD28, ICOS, and HER-2 were designed in pcDNA3.1 expression vector (Life Technologies) and sourced from Genscript, USA. DNA was introduced into the cell using the Expi293F transient transfection system (Life Technologies) as described above and incubated for 48 h. For analysis by flow cytometry, 200,000 transfected or negative control were plated in 96 well round bottom plates. Cells were spun down and resuspended in staining buffer [PBS (phosphate buffered saline), 1% BSA (bovine serum albumin), and 0.1% sodium azide] for 20 min to block non-specific binding. Afterwards, cells were centrifuged again and resuspended in staining buffer containing 100 nM to 50 pM V-mAb or control protein in 50 μl. Primary staining was performed on ice for 45 min, before washing cells in staining buffer twice. PE-conjugated anti-human Fc (Jackson ImmunoResearch, USA) was diluted 1:150 in 50 μl staining buffer and added to cells and incubated another 30 min on ice. Secondary antibody was washed out twice, cells were fixed in 2% formaldehyde/PBS, and samples were analyzed on a LSRII (Becton Dickinson, USA) or a Hypercyt flow cytometer (Intellicyte, USA). Mean Fluorescence Intensity (MFI) was calculated for each transfectant and negative parental line with Cell Quest Pro software (Becton Dickinson, USA) or Forcyte software (Intellicyt, USA).

Costimulatory activity of V-mAbs was determined in anti-CD3 coimmobilization assays. Ten nanomolars of mouse anti-human CD3 (OKT3, Biolegend) was diluted in PBS with V-mAbs over a range of concentrations from 40 to 0.625 nM or control proteins and added to tissue culture treated flat bottom 96 well plates (Corning, USA) for overnight incubation. Unbound protein was washed off the plates with PBS and 100,000 purified human purified human T cells (BenTech Bio, USA) labeled with 0.25 uM CFSE were added to each well in a final volume of 200 μl of Ex-Vivo 15 media (Lonza, Switzerland). Cells were cultured 3 days and cell culture supernatants were analyzed for human IFN-gamma production by ELISA (Duoset ELISA kit, R&D Systems). Cellular proliferation was determined by CFSE dilution on cells stained with fluorescently-conjugated anti-CD4, anti-CD8 antibodies (BD, USA) or total T cells via flow cytometric analysis on an LSR II (BD, USA).

For cytokine responses to tumors with endogenous levels of HER-2, 100,000 human T cells (Bentech) were plated with 25,000 NCI-N87-OKT3 (HER2+) human gastric carcinoma with 30nM V-mAbs or control proteins. Supernatants were assayed 72 h by ELISA for IFN-γ production by ELISA.

Human CD3+ T-cells were transduced with lentiviral vector encoding the HLA-A^*^0201 restricted TCR directed against the E6 epitope of the viral oncoprotein human papillomavirus type 16 (HPV-16). Parental or HER-2-transduced CEM.T2 cells were pulsed with 1 ng ml^−1^ E6 peptide (29–38) for 90 min then washed. E6 TCR T cells were plated at a effector to target (E:T) ratio of 1:3. V-mAbs were titered from 30 nM to 300 pM and incubated with effector and target cells. Supernatants were harvested for measurement of IFN-γ secretion by ELISA after 24 h.

### Delayed Type Hypersensitivity (DTH) Model

Female 8-week-old BALB/cN mice (Charles River Laboratories) were weighed, divided into groups with similar mean and body weight (BW) range, and then injected subcutaneously (SC) at the base of the tail on day 0 with 100 μg low endotoxin chicken ovalbumin (OVA) (Worthington Biochem) emulsified in 100 μL Sigma Adjuvant System (Sigma). On day 7, mice were treated with PBS or one of the test articles via intraperitoneal (IP) injection (*n* = 7 mice per treatment group). ICOSL vIgD-Fc or abatacept (CTLA4-Fc; Orencia^TM^; Catalent Pharma Solutions) test articles were kept frozen at −80°C until just prior to dilution for dosing with PBS. ICOSL vIgD-Fc proteins were dosed at 150 μg per animal and abatacept at 112 μg with these two doses providing molar equivalent doses of the different proteins. Two to three hours after dosing with test articles, each mouse was anesthetized with isoflurane gas and baseline ear thickness was measured using Mitutoyo calipers (Sakado). The mice were then challenged with 10 μg of OVA in 10 μL PBS via intradermal (ID) injection into the left ear pinnae with the uninjected right ear serving as a negative control. Ear thickness was again measured for each mouse under isoflurane on day eight. Differences in the mean change in ear thickness between treatment groups were determined using a 1-way analysis of variance (ANOVA) statistical analysis.

### Human PBMC NSG Mouse Model of Graft vs. Host Disease (GvHD)

NOD.Cg-Prkdc<scid> Il2rg <tm1Wjl>/SzJ (NSG^TM^; JAX) female mice aged 6–8 weeks old were grouped per body weight into treatment groups on day −1, irradiated with 1 Gy from an X-ray irradiator source, and administered 10 mg gamma globulin (human IgG, Sigma) SC. On study day 0 (within 24 h post-irradiation), mice were dosed IP with 100 μL saline, 75 μg belatacept (Nulojix^TM^; Catalent Pharma Solutions), or 100 μg of WT ICOSL-Fc or ICOSL vIgD-Fc proteins (*n* = 9 mice per treatment group). All mice received 10^7^ human PBMCs injected intravenously (IV) on day 0. Dosing with test articles continued 3 times per week from day 0 through day 37, and surviving mice were terminated on day 49. Cage side observations were made daily and body weight (BW) and clinical observations were performed twice weekly. Once mice demonstrated clear clinical signs, clinical observations were made daily. Mice were assessed for BW loss and a disease activity score (overall health and activity, skin and hair changes, and BW loss). Mice were euthanized by CO_2_ asphyxiation before study termination if they showed >20% BW loss from their starting weight or a combination of the following clinical signs: >10–20% BW loss from their starting weight, cold to touch, or lethargic, pale, hunched posture, and scruffy coat. The mean DAI scores were plotted for the time course of the experiment, with the last observation (i.e., mean scores collected on day of termination) carried forward on the graph for those groups terminated prior to the last planned study day (Day 49). Significant differences among groups for data over time (i.e., DAI scores) were determined using 2-way repeated measures ANOVA for treatment effects. To determine statistical differences in survival proportions among groups, data were analyzed using the Mantel-Cox (log-rank) test. The datasets generated during and/or analyzed during the current studies are available from the corresponding author on reasonable request.

### Ethical Approval

All animal procedures were approved by the appropriate Institutional Animal Care and Use Committee overseeing the vivarium where the studies were conducted (Alpine Immune Sciences and The Jackson Laboratory), and followed the guidelines set forth in the 8th Edition of the Guide for the Care and Use of Laboratory Animals (National Research Council, 2011).

## Results

### Directed Evolution of ICOSL Domains

**Figure 1:**
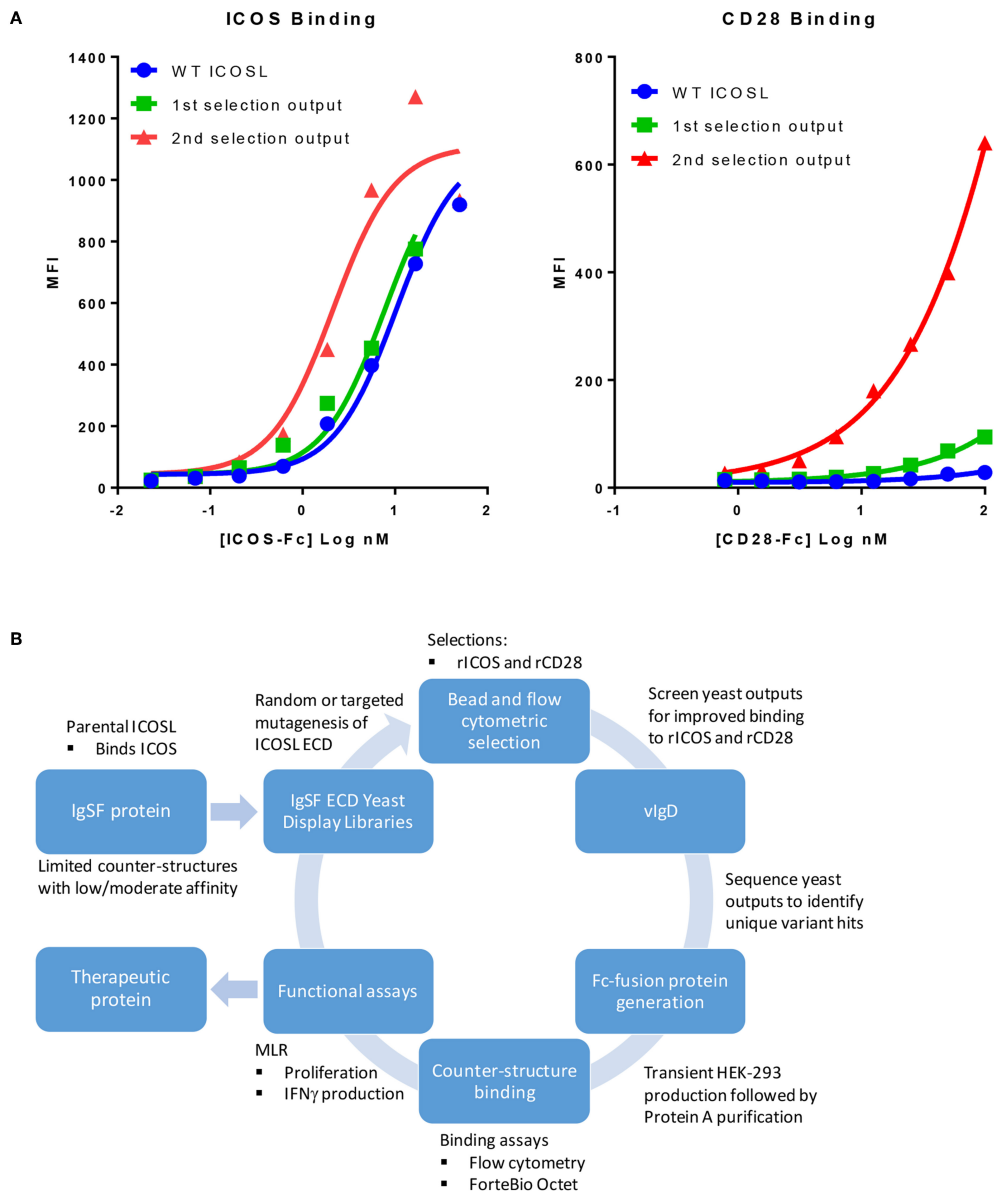
Our scientific platform can yield yeast population outputs with increased affinity for cognate ligands and/or new binding partners. **(A)** Selections with two ligands results in yeast display of engineered ICOSL domains with increased binding toward both counter-structures. Yeast were transformed with a 1st generation random engineered ICOSL domain library and affinity matured by selection with indicated recombinant receptors. Individual receptor binding to bulk yeast populations by flow cytometry are shown. Improvements to binding against both receptors were noted at each selection. **(B)** Schematic showing yeast display strategy for selection and characterization of engineered vIgDs with altered specificity and/or affinity.

We sought to develop a platform that would allow us to generate libraries of IgSF proteins, each with a limited number of mutations with the potential to alter the affinity of native and/or non-native interactions. To do this, libraries of ICOSL were developed using error prone PCR to introduce between 1 and 3 random mutations per clone. Libraries of independent clones were then used to generate yeast expression libraries that allowed for surface display of the ICOSL variants. This library DNA was introduced into yeast and the resulting yeast populations were subjected to two rounds of bead-based selection and two rounds of FACS-based sorting using recombinant Fc-fusion proteins for detection of binding to displayed variants. Selections were carried out with alternating rounds of sorting using recombinant ICOS-Fc followed by selections using recombinant CD28-Fc. By using progressively lower protein concentrations for each round of selection, yeast populations were identified that displayed ICOSL variants with increased affinity for the target proteins (Figure 1A). Because the affinity of native ICOSL for ICOS is relatively high compared to the affinity of native ICOSL to CD28, the differential of increased binding affinity per round of selection was more modest than the increase in CD28 binding, which initially bound ICOSL very poorly (Figure 1A) (13). Selections for elevated CD28 binding concomitantly yielded elevated binding to CTLA4 as well, despite the fact there was no specific selection in favor of this interaction. This observation perhaps suggests selection for variants that more effectively interacted with the highly conserved sequence in CTLA4 and CD28 responsible for binding to the ICOSL-related proteins CD80 and CD86 (14). DNA was isolated from the selected yeast populations and a group of unique ICOSL clones each with a varying number of amino acid substitutions were isolated and cloned into mammalian expression vectors such that the encoded proteins could be expressed in mammalian cells as Fc-fusion proteins. Individual coding elements were then sequenced and screened for binding to HEK-293 cells that had been transiently transfected with either ICOS or CD28 using flow cytometry. Yeast outputs that were screened in this way that showed reasonable sequence diversity and acquisition of the desired binding properties were then subjected to additional rounds of mutagenesis, selection, and screening as described above. Selections for ICOSL variants went through three rounds in the selection process with individual clones isolated and characterized from each generation. The resultant proteins were termed ICOSL vIgD-Fc proteins and the entire process for generating them is diagrammed in Figure 1B.

**Figure 2:**
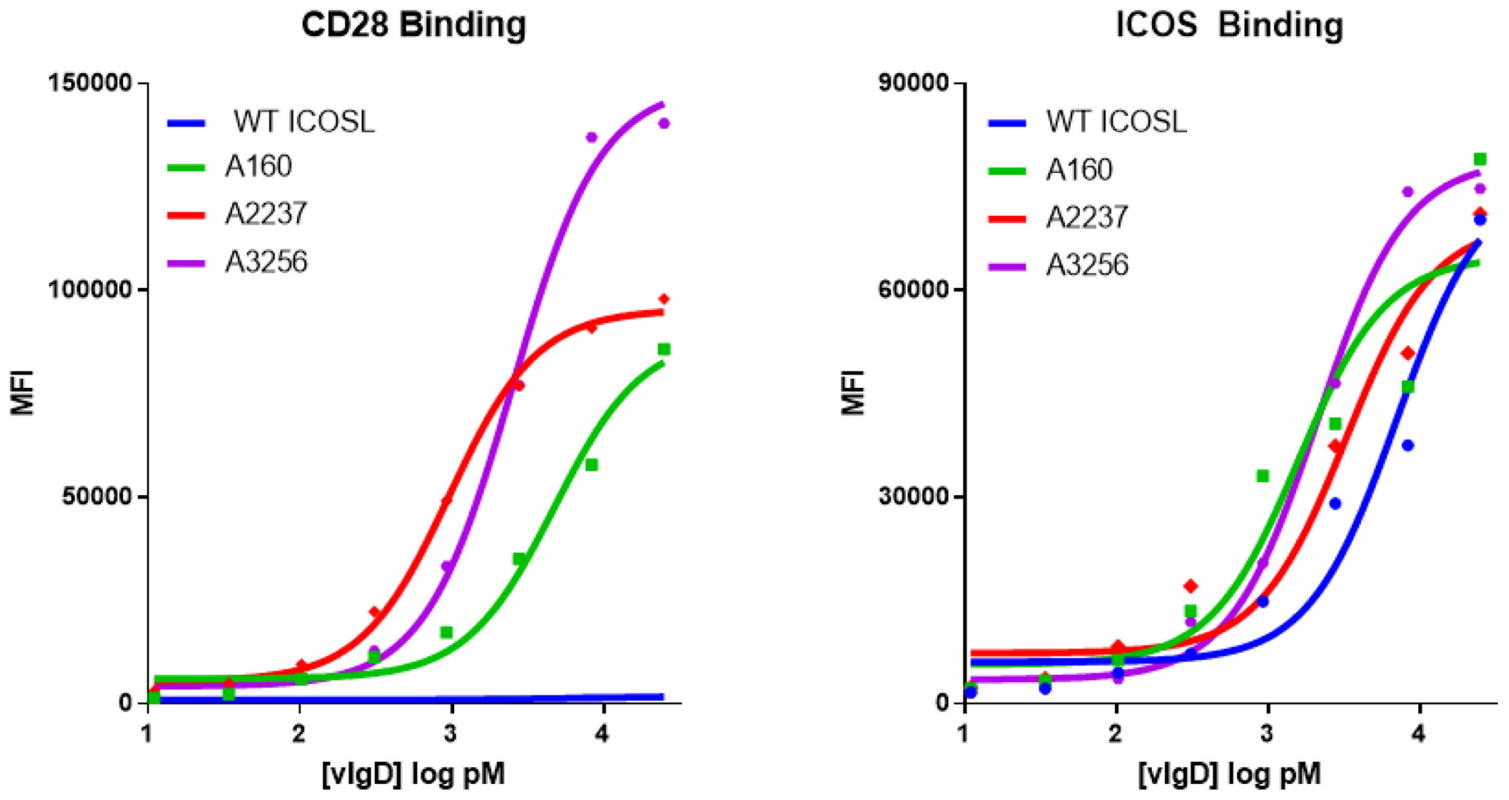
Directed evolution of ICOSL by yeast display leads to recombinant vIgD hits with enhanced binding to ICOS and CD28. ICOSL DNA sequences derived from yeast display outputs were cloned into a mammalian expression vector and produced in HEK-293 cells. Engineered ICOSL vIgD-Fc were subsequently titrated on CD28 **(left)** and ICOS **(right)** transfectants. Binding was detected by flow cytometry and Mean Fluorescence Intensity (MFI) was plotted vs. protein concentration. For comparison, ICOSL vIgD-Fc derived from 1st generation (A160), 2nd generation (A2237), and 3rd generation (A3256) are compared to WT ICOSL, showing progressive increases in binding for each target.

Individual engineered ICOSL domains obtained from the iterative yeast selections and sorts were expressed as Fc-fusion proteins and more detailed binding analysis was carried out. Purified protein was quantified and bound to HEK-293 cells transiently transfected with ICOS or CD28 over a range of ICOSL vIgD-Fc protein concentrations. Bound proteins were detected by flow cytometry and the level of binding was quantitated from the mean fluorescence intensity (MFI). The resultant binding curves were used to generate EC_50_ values. Figure 2 shows binding curves for several ICOSL vIgD-Fc, representative of outputs from 1st generation (A160), 2nd generation (A2237), and 3rd generation (A3256) selections. In addition, ICOSL vIgD-Fc were tested for binding to both counter-structures using ForteBio (Table 1). Although reported dissociation constants reflect avidity effects from measuring binding affinities with dimeric Fc-fusion proteins, sub-nanomolar K_D_ values against both counter-structures were demonstrated.

**Table 1:** ICOSL vIgD-Fc exhibit increased affinities against ICOS, CD28, and CTLA4.

	**ICOS**		**CD28**		**CTLA-4**	
**Sample ID**	**KD**	**Fold imp**.	**KD**	**Fold imp**.	**KD**	**Fold imp**.
WT ICOSL	883.4 pM		13.88 nM		77.12 nM	
H160	331.5 pM	2.7	524.7 pM	26.5	677.6 pM	113.8
H180	337.9 pM	2.6	1.163 nM	11.9	1.522 nM	50.7
H183	769.1 pM	1.1	782.9 pM	17.7	832.8 pM	92.6
H184	381.7 pM	2.3	435.9 pM	31.8	646.8 pM	119.2
H2227	1.294 nM	0.7	896 pM	15.5	1.466 nM	52.6
H2229	491.8 pM	1.8	447.2 pM	31.0	644.9 pM	119.6
H2231	372.7 pM	2.4	400.5 pM	34.7	591.7 pM	130.3
H2236	471.6 pM	1.9	389.9 pM	35.6	575.9 pM	133.9
H2237	420.3 pM	2.1	293.4 pM	47.3	535.6 pM	144.0
H2239	368.6 pM	2.4	367.9 pM	37.7	719.4 pM	107.2
H2241	336.8 pM	2.6	1.042 nM	13.3	2.518 nM	30.6
H3256	542.8 pM	1.6	502.6 pM	27.6	1.059 nM	72.8
H3269	362.2 pM	2.4	553 pM	25.1	647.9 pM	119.0
H3305	477 pM	1.9	562.9 pM	24.7	1.066 nM	72.3
H3310	477.1 pM	1.9	365.9 pM	37.9	864.3 pM	89.2
H3318	339.8 pM	2.6	968.7 pM	14.3	1.122 nM	68.7
H3321	371.3 pM	2.4	1.947 nM	7.1	2.812 nM	27.4
H3322	310.6 pM	2.8	909.8 pM	15.3	1.2 nM	64.3

*Protein-protein interactions between the receptors and ICOSL variants were assessed using ForteBio Octet binding assays. Human recombinant ICOS-Fc CD28-Fc, or CTLA4-Fc fusion proteins were loaded individually onto anti-human capture sensors (ForteBio Octet AHC) and wild type human ICOSL-Fc fusion protein or ICOSL vIgD-Fc were bound to the receptors in four-point titrations (100, 50, 25, and 12.5 nM). Each titration was globally fit with a 1:1 model using the ForteBio Analysis software to calculate the association and dissociation rates of each protein (data not shown). The dissociation constant (K_D_) was then calculated and compared to wild type ICOSL to determine a fold improvement value*.

### Mapping ICOSL Mutations Selected for Increased ICOS/CD28 Binding

**Figure 3:**
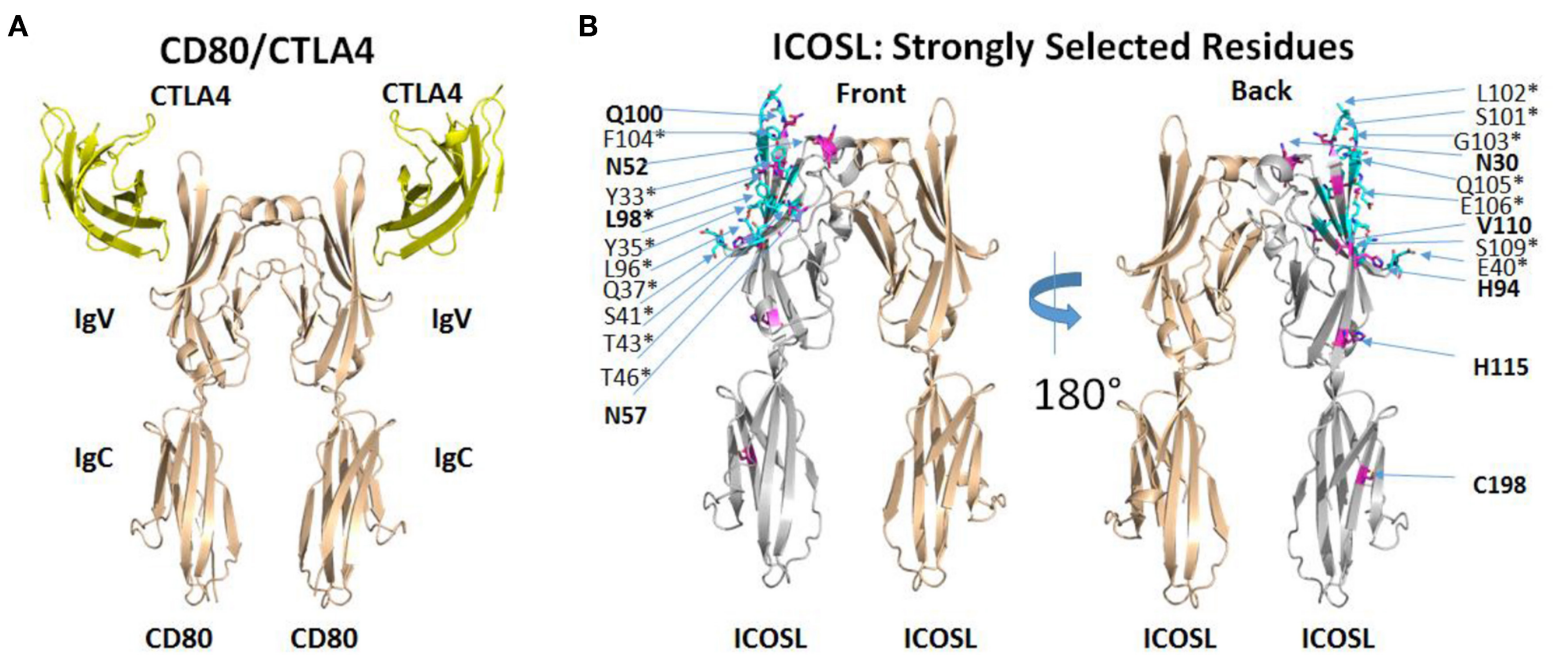
ICOSL mutations identified at predicted interaction sites of homologous B7/CD28 family members. **(A)** Interaction of human CD80 with human CTLA4 as deduced from the crystal structure of CD80 extracellular domain dimer complexed with single extracellular domain of CTLA4 per CD80 monomer (PDB ID: 1I8L). **(B)** Homology model of human ICOSL extracellular domain based on CD80/CTLA4 complex. ICOSL residues predicted to be located within 5 angstroms of ICOS are colored cyan and marked by an asterisk. ICOSL residues frequently mutated through yeast library selection are colored purple and indicated in bold. Residues are split between front and back view of ICOSL dimer (rotated 180°) to maximize visibility in figure.

The selection of ICOSL variants with increased affinity for both ICOS and CD28 revealed several amino acid substitutions that appeared to be conserved across multiple clones (Supplementary Table 1) and appeared to be limited to the ICOS/CD28 binding IgV domain of ICOSL. Some substitutions showed up less regularly, suggesting that these might be less critical in directing binding properties. Therefore, we mapped recurring mutations onto the structure of ICOSL. There is no published structure for the ICOSL/ICOS complex, but an alignment of CD80 with ICOSL based on a homology model of ICOSL/ICOS templated on the structure of the CD80/CTLA4 complex has been previously described (15). To better understand why mutations selected by yeast display improved binding of both ICOS and CD28, we used PDB ID 1l8L as a template to generate an analogous three-dimensional homology model to map the mutations directly on the ICOSL structure (Figure 3; see section Methods). Except for C198, frequently observed mutations in clones derived from selections for increased CD28 and ICOS binding are confined to the IgV domain of ICOSL (see Figure 3, left panel for location of CTLA4 as proxy for position of ICOS and compare to right panel). In contrast, residues found to be less frequently mutated in selected variants are distributed across ICOSL IgV and IgC domains with some bias toward the IgC domain (data not shown). With display technologies such as yeast display, the frequency of selection of a mutated residue generally correlates with likelihood of impact on binding (16). The results, therefore, suggest recurrently mutated residues in the IgV domain affect the ICOSL/ICOS interaction, while mutations in the IgC domain are less frequent across different clones and thus are more likely non-functional “bystander” mutations that are products of library generation by random mutagenesis. Only the recurrent mutation at position 98 affects an ICOSL amino acid residue within 5 angstroms of the predicted ICOSL site of interaction with ICOS. The only other mutated residue within 5 angstroms of the ICOS binding site is at position 96 and these mutations are only infrequently observed across clones. However, the proximity of residues 96 and 98 suggests residue 96 may in fact be another residue that is part of the binding interface. Consistent with this notion, CD80 residues structurally equivalent to residues 96 and 98 are contact residues for CTLA4 (17). Strongly selected mutations at positions other than position 96 (and perhaps 98) in ICOSL are not predicted to be in the predicted ICOS-binding interface. In the absence of direct structural information, the precise contributions of these other mutations to binding is speculative, but it seems reasonable to postulate that they modulate ligand binding indirectly through allosteric effects transmitted through the IgV domain and into the interface with ICOS as predicted from the modeled structure (Figure 3). Notably, residue 52 is predicted to be glycosylated and removal of a glycan through the mutation of the asparagine may impact binding through alteration of ICOSL tertiary structure. It is therefore implied that yeast display selection mostly yielded novel solutions for improving binding, rather than through improving actual contact residues. Overall, the results suggest the most strongly selected mutations affect the ICOS interface either directly or indirectly. This interface is analogous to the CD80/CTLA4 interface (15) and it is well-established that the CD28 binding epitope on CD80 overlaps the CTLA4 binding epitope and both ligands utilize similar sequence features to contact CD80 (17). Results from this structure/function analysis therefore provide an explanation why improved binding of ICOS is associated with improved binding to CD28 even though mutants were selected with either ICOS-Fc or CD28-Fc as selection agents.

### Engineered ICOSL vIgD Antagonize T Cell Activation *in vitro*

To assess the biological impact of increased affinity for both CD28 and ICOS, more than 100 independent ICOSL vIgD-Fc reflecting a range of affinities for CD28, ICOS, and CTLA4 (Table 1, data not shown) were tested in a human MLR functional assay and effects on T cell responses were compared to reagents antagonizing only CD28 signaling (CTLA4-Fc) or only ICOS signaling (WT ICOSL-Fc). Specifically, monocyte derived dendritic cells (DCs) from one donor were used to stimulate purified T cells from an MHC mismatched donor to generate an allogeneic T cell response. T cell responses were assessed by monitoring release of interferon-gamma (IFNγ). Test articles including WT ICOSL-Fc, CTLA4-Fc, and the ICOSL vIgD-Fc were initially tested over a narrow range of concentrations to assess their impact on the MLR. The CTLA4-Fc variant used in these assays was the clinically approved therapeutic protein belatacept, which harbors two point mutations resulting in an increased affinity for the CD28 ligands CD80 and CD86 relative to wild-type CTLA4 (18, 19). These initial screenings demonstrated belatacept inhibited IFNγ production in MLR assays while WT ICOSL-Fc minimally affected this parameter. However, many of the ICOSL vIgD-Fc inhibited production of IFNγ more effectively than belatacept (data not shown).

**Figure 4:**
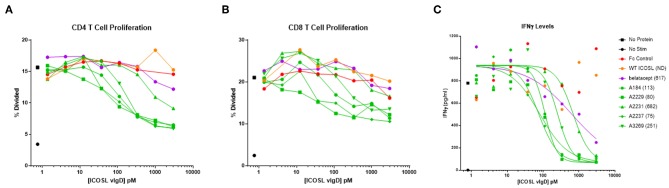
Soluble ICOSL vIgD-Fc effectively attenuate T cell responses in mixed lymphocyte reaction (MLR). Monocyte derived dendritic cells from one donor were used to allogeneically stimulate purified T cells from another donor. Responses were measured by assessing **(A)** CD4 T cell proliferation, **(B)** CD8 T cell proliferation, and **(C)** IFNγ levels in culture supernatants. Proliferation results use CFSE dilution to show the percentage of divided cells vs. protein concentration. IFNγ levels are shown as pg ml^−1^ vs. protein concentration, with IC_50_ pM values calculated using GraphPad Prism and shown in parenthesis on graph label. ND, not determined. Results shown are representative of at least three separate experiments performed with each protein.

To better quantify the relative potencies of ICOSL vIgD-Fc compared to belatacept and WT ICOSL-Fc, 16 of the most potent ICOSL vIgD-Fc (as determined from MLR screening assays described above) were re-tested in an MLR over a broad range of lower concentrations. In this analysis, responses were measured by assessing proliferation and IFNγ production. Belatacept proved to be much more potent than WT ICOSL-Fc at inhibiting proliferation (Figures 4A,B). This result is consistent with the fact that CD28 is constitutively expressed on T cells, whereas ICOS must be induced by an initial activation event before its signaling can become relevant (20). All ICOSL vIgD-Fc tested inhibited T cell proliferation more effectively than WT ICOSL-Fc and many were superior to belatacept (Figures 4A,B). Expression analysis by flow cytometry confirmed that more than 95% of CD4+ T cells and 65–80% of CD8+ T cells expressed CD28 in all donors prior to stimulation while there was very little ICOS staining (data not shown). However, at the termination of the assays, while CD28 staining was relatively unchanged, ICOS staining was increased and the cells that expressed ICOS corresponded to those that had proliferated as determined from CFSE dilution (data not shown).

Production of IFNγ was also studied. IC_50_ values were determined from the IFNγ release data and results for five ICOSL vIgD-Fc proteins are shown in Figure 4C. Based on these calculations, all ICOSL vIgD-Fc proteins out-performed WT ICOSL-Fc and most were superior to belatacept as well, although the magnitude of the inhibition varied (Figure 4C). This analysis revealed ICOSL vIgD-Fc proteins A2237, A2229, and A184 (Figure 4C; Table 1) were the most effective in inhibiting IFNγ release in an MLR.

To confirm that binding of the ICOSL vIgD-Fc proteins correlated with inhibitory activity, cells were stained at the termination of the assay with fluorochrome conjugated anti-human IgG antibody to detect Fc-fusion proteins bound to the T cells, and the level of binding was quantified based on MFI. There was a strong correlation between the level of binding and the magnitude of the inhibitory response in these assays (Supplementary Figure 2).

Since the engineered ICOSL variants also bound CTLA4 in addition to ICOS and CD28, we attempted to evaluate whether this interaction had any impact on responses in the MLR. To evaluate this possibility, MLR assays were done in the presence or absence of the blocking anti-CTLA4 antibody ipilimumab (21). Inclusion of a saturating level of ipilimumab (30 nM) did not significantly impact responses in the MLR regardless of whether an Fc control protein or an engineered ICOSL vIgD-Fc protein was included (Supplementary Figure 3a) indicating that CTLA4 does not play a significant role in this particular assay system and supporting the conclusion that ICOSL vIgD-Fc proteins inhibit responses primarily by blocking costimulation through CD28 and ICOS.

As discussed above, most primary resting T cells fail to express ICOS although it is rapidly upregulated with activation. To test the effect of ICOSL vIgD-Fc proteins on T cells expressing both ICOS and CD28, primary human T cells were stimulated for 2 days with anti-CD3 and anti-CD28 antibodies and expression of CD28 and ICOS were examined. More than 75% of T cells stimulated this way co-expressed both ICOS and CD28 after stimulation. These T cells were then stimulated for a second time with K562 cells expressing a single chain Fv version of the anti-CD3 antibody OKT3 to provide antigen receptor stimulation and either ICOSL alone, or the combination of ICOSL, CD80, and CD86. ICOSL vIgD-Fc proteins were included over a range of concentrations and effects on T cell proliferation were evaluated. T cell responses were inhibited less effectively in this secondary stimulation, but inclusion of ICOSL vIgD-Fc A2237 was still able to inhibit responses and did so much more robustly than either of the CD28-pathway antagonists abatacept or belatacept for both proliferative and cytokine responses (Supplementary Figures 2b,c). As expected, belatacept and abatacept were more effective antagonizing responses to K562 that were also transduced with CD80 and CD86. The slight decrease in responses to K562 cells expressing only ICOSL are likely due to low levels of CD80 expressed on K562 cells (data not shown), but clearly in both cases the dual antagonist A2237 ICOSL vIgD-Fc was more effective than antagonists of either CD28 or ICOS pathways alone (Supplementary Figures 2b,c).

### Engineered ICOSL vIgD Antagonize T Cell Activation *in vivo*

To determine if the functional activity established for the ICOSL vIgD-Fc proteins *in vitro* translated *in vivo*, experimental mouse models were explored allowing use of these human proteins *in vivo*. ICOSL vIgD-Fc proteins cross-reacts with mouse ICOS and CD28 to varying degrees (Supplementary Table 2). Three ICOSL vIgD-Fc (A2237, A2231, and A2229), each with varying degrees of efficacy in the MLR (Figure 4), were selected for testing *in vivo*, in comparison to WT CTLA4-Fc (abatacept) which is also effective in mice with good binding to mouse CD80 and CD86 (Supplementary Table 2).

**Figure 5:**
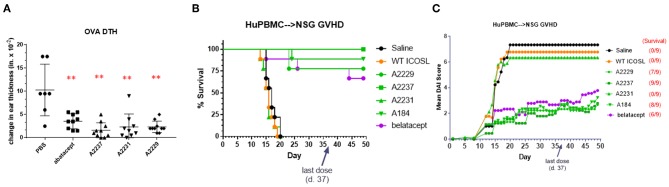
ICOSL vIgD-Fc suppress immune responses *in vivo*. **(A)** A delayed type hypersensitivity (DTH) model was performed by sensitizing mice with OVA and subsequently rechallenging with OVA in the ear pinna. Groups of seven mice treated with either abatacept or ICOSL vIgD-Fc showed significantly less OVA-induced ear swelling as compared to PBS treated animals (^**^p < 0.0001 by 1-way ANOVA). Bars shown are the group mean (s.d.). **(B,C)** An acute model of graft-versus-host-disease (GvHD) was performed by adoptively transferring human PBMC into immunodeficient NSG mice (*n* = 9/group). **(B)** High affinity ICOSL vIgD-Fc significantly prolonged survival and **(C)** significantly reduced mean disease activity index (DAI). Administration of ICOSL vIgD-Fc protected from effects of GvHD at levels comparable to or better than belatacept, but wild-type ICOSL-Fc or lower affinity ICOSL vIgD-Fc were not effective in protecting from GvHD in this model. For **(B)**, by log-rank test, belatacept and high affinity ICOSL vIgD-Fc significantly prolonged survival as compared to saline and WT ICOSL-Fc (*p* < 0.001) treatments; ICOSL vIgD-Fc A2237 prolongs survival as compared to belatacept (*p* = 0.065). For **(C)**, by 2-way repeated-measures ANOVA, belatacept and high affinity ICOSL vIgD-Fc significantly reduce DAI scores as compared to saline and wild-type ICOSL-Fc (*p* < 0.001); ICOSL vIgD-Fc A2229 and A2237 were significantly better at reducing DAI scores than belatacept (*p* = 0.053 and *p* = 0.035 for A2229 and A2237, respectively). Both studies were performed at least twice with representative experiments shown here.

The first model in which ICOSL vIgD-Fc proteins were evaluated was a short-term model of T cell-mediated inflammation, an ovalbumin (OVA)-based delayed type hypersensitivity (DTH) reaction. Mice were sensitized by subcutaneous injection of OVA followed 7 days later by intradermal OVA challenge in the ear pinnae. Animals were treated with test articles just prior to challenge using doses that approximated the 5 mg kg^−1^ dosing representing a mid-range of recommended dosing for abatacept and the corresponding molar equivalent of the ICOSL vIgD-Fc proteins (see section Methods). Ear swelling was monitored several hours later to assess inflammation. Administration of abatacept and all ICOSL vIgD-Fc proteins tested significantly reduced ear swelling in treated animals compared to PBS treated controls (Figure 5A). These data demonstrate recombinant human ICOSL vIgD-Fc can attenuate an immune response *in vivo* in this acute challenge model.

To examine the effects of the ICOSL vIgD-Fc in a more prolonged inflammation model, the same ICOSL vIgD-Fc proteins plus an additional variant, A184, were tested in a huPBMC-NSG™ model of graft-versus-host disease (GvHD). Human PBMC were transferred into NSG mice, which induces an acute xenogeneic GvHD response (22). Engrafted mice were treated with wild type ICOSL-Fc, ICOSL vIgD-Fc, belatacept, or saline as a negative control. Survival and disease index scores were monitored as described in Methods. Treatment with A2237, A2229, and A184 ICOSL vIgD-Fc significantly enhanced survival (Figure 5B) and attenuated disease development (Figure 5C) in this model, while WT ICOSL-Fc and A2231 ICOSL vIgD-Fc, which had reduced activity in the MLR *in vitro* (Figure 4), did not. In fact, A2237, A2229, and A184 ICOSL vIgD-Fc protected animals from developing a GvHD response as well or better than belatacept (Figures 5B,C). A2237, A2229, and A184 ICOSL vIgD-Fc also conferred significantly greater disease protection than the WT ICOSL-Fc, demonstrating the enhanced biological activity of engineered proteins generated through the vIgD platform. These data indicate ICOSL vIgD-Fc can protect animals from development of GvHD in this human PBMC-mouse model, and that the activity of the most potent antagonist ICOSL vIgD-Fc tested (A2237) is more protective than belatacept in this model.

### ICOSL vIgD Costimulate T Cells When Tethered to a Surface

**Figure 6:**
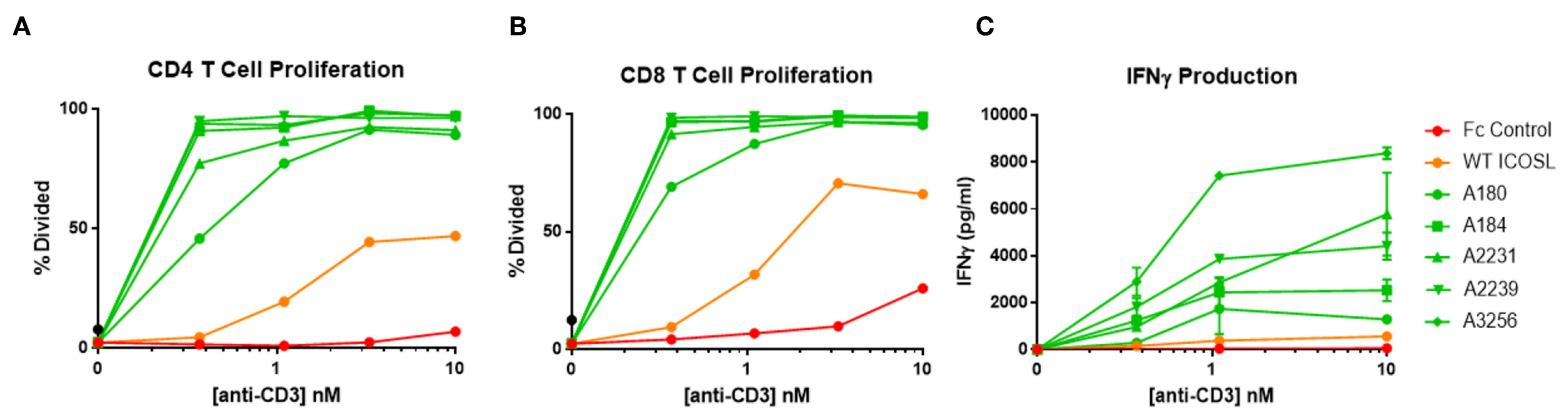
Immobilized, engineered ICOSL-Fc variants costimulate T cells when plated with sub-optimal anti-CD3. Human T cells were stimulated with a range of anti-CD3 concentrations and a constant concentration of coimmobilized ICOSL-Fc variants (40 nM). **(A)** CD4 and **(B)** CD8 T cell proliferation was monitored by CFSE dilution. Proliferation is reported as percentage of T cells divided vs. anti-CD3 concentration. **(C)** IFNγ production was measured by ELISA in supernatants collected at 72 h and plotted as pg/ml cytokine vs. anti-CD3 concentration. Each point represents the mean of triplicate determinations ±s.d.

ICOSL vIgD-Fc were also tested for their capacity to costimulate T cells when immobilized on a surface to mimic the cell surface clustering normally required for agonist activity. In most cases, inclusion of plate-coated ICOSL vIgD-Fc with sub-optimal levels of anti-CD3 antibody resulted in dramatically enhanced T cell responses compared to anti-CD3 plus control Fc-proteins or WT ICOSL-Fc. This costimulatory effect was dependent on antigen receptor engagement since stimulation with plate bound ICOSL-Fc proteins in the absence of anti-CD3 antibody failed to activate T cells in these assays (data not shown). Specifically, proliferation of CD4+ (Figure 6A) and CD8+ T cells (Figure 6B) and release of IFNγ (Figure 6C) were dramatically enhanced when plate-bound ICOSL vIgD-Fc were co-immobilized with anti-CD3 antibody.

**Figure 7:**
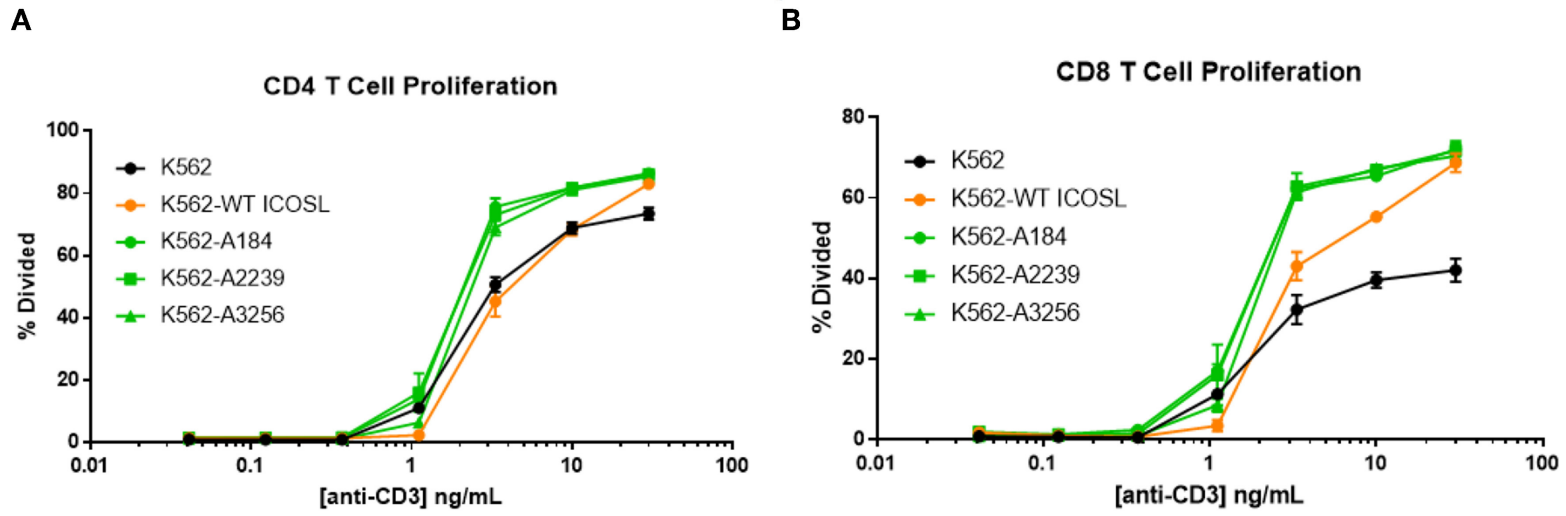
ICOSL vIgD-Fc expressed on the surface of cells deliver a costimulatory T cell signal. Cell Trace Far Red labeled human T cells were cocultured with K562 cells expressing the indicated versions of ICOSL vIgD-Fc and stimulated with a range of soluble CD3 antibody concentrations. Cells were harvested after 72 h and proliferation of **(A)** CD4 and **(B)** CD8 human T cells is reported as percent of cells divided vs. anti-CD3 concentration. Each point represents the mean of triplicate wells with error bars showing standard deviation (s.d.).

The capacity of these ICOSL vIgD-Fc to deliver a costimulatory signal when anchored to a surface led us to explore alternative mechanisms for achieving this selective costimulation of T cell activation. The first strategy tested was to express the engineered ICOSL domains on the surface of the K562 cell line by fusing the ECD of the engineered ICOSL domains to the native transmembrane and intracellular domain to generate a Transmembrane Immunomodulatory Protein (TIP), essentially creating a cell-bound engineered ICOSL domain with enhanced binding to ICOS and CD28. To provide a TCR signal for T cells, anti-CD3 antibody was included in soluble format over a range of concentrations allowing K562 presentation of this stimulating antibody through the Fc-receptor CD32 expressed by the cells. Wild type K562 cells stimulated T cells to proliferate when co-incubated with soluble anti-CD3 antibody in a dose-dependent manner, whereas K562 cells in the absence of anti-CD3 did not (Figures 7A,B). Expression of WT ICOSL TIP on the surface enhanced responses somewhat (Figures 7A,B), but the effects were much more dramatic when K562 cells expressed TIPs containing engineered ICOSL domains, indicating these ICOSL vIgD TIPs can provide superior costimulation for T cells when expressed on the surface of cells.

### Delivery of Localized T Cell Costimulation via Tumor Localizer

One application for utilizing the costimulatory properties of these engineered ICOSL domains could be to direct them to tumors to provide a costimulatory signal to resident, tumor-specific T cells in malignant diseases. Specific localization of a costimulatory molecule would potentially limit T cell activation to sites where that costimulatory signal would be most beneficial and restrict activation systemically in organs and tissues where T cell activation could have deleterious effects.

**Figure 8:**
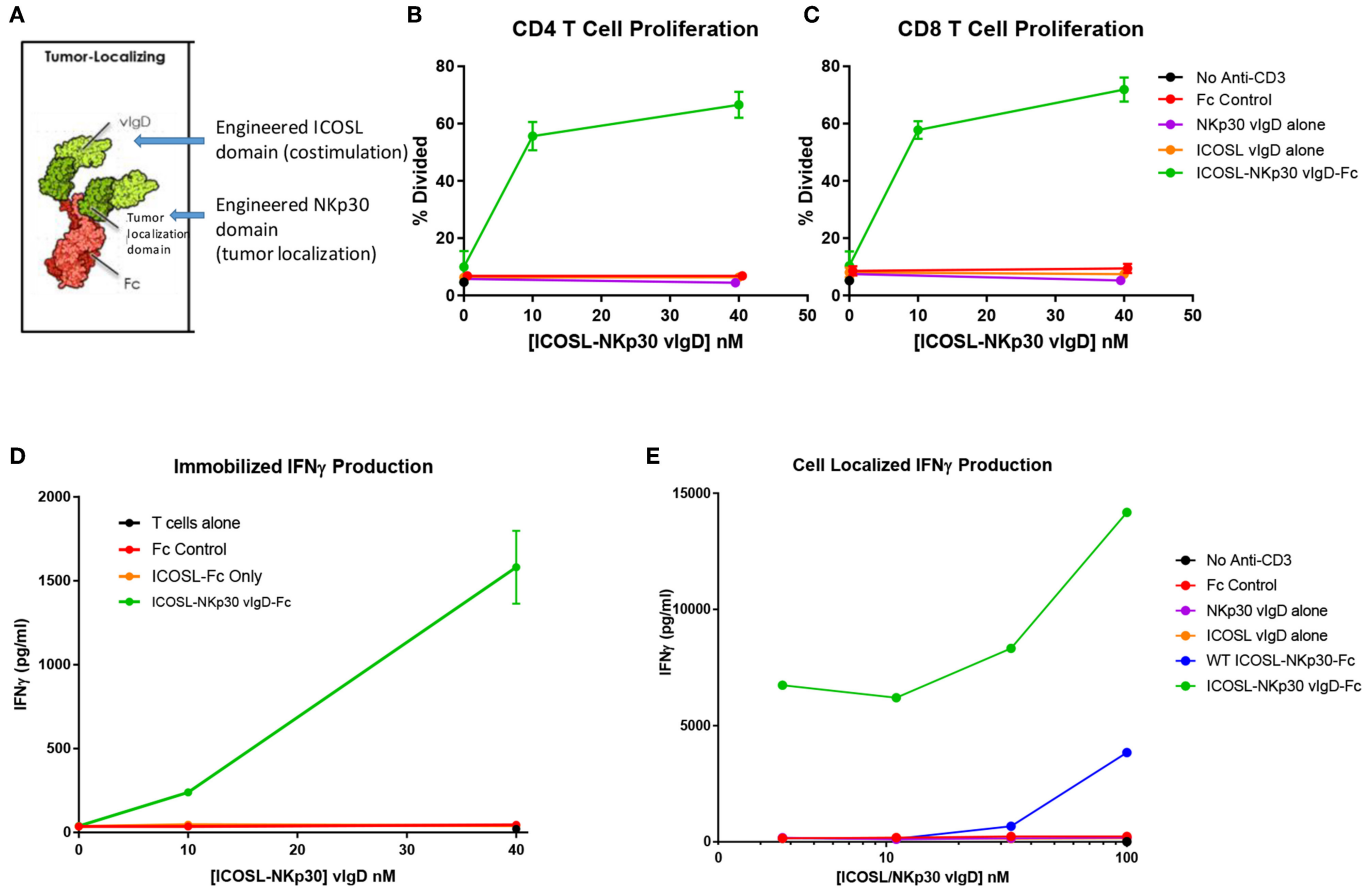
ICOSL vIgD proteins can be formatted to provide localized T cell costimulation. **(A)** Schematic diagram of a tumor localizing fusion protein consisting of an ICOSL vIgD costimulatory domain (N-terminal), a variant NKp30 domain to localize the protein to B7H6 expressing tumors, and an antibody Fc domain. **(B–D)** ICOSL-NKp30 vIgD-Fc fusion proteins can provide T cell costimulatory signals that are dependent on the presence of B7H6. Plates were coated with 40 nM recombinant B7H6-Fc protein and 10 nM anti-CD3. CFSE-labeled primary human T cells were added with titrated concentrations of the variant ICOSL-Fc alone (orange circle), the variant NKp30-Fc alone (purple circle), or an ICOSL-NKp30-Fc variant fusion protein (green circles) for 3 days. Cells were analyzed for proliferation by CFSE dilution in **(B)** human CD4 or **(C)** human CD8 T cells. **(D)** Supernatants were collected and assessed for IFNγ production by ELISA. **(E)** Variant ICOSL-NKp30-Fc fusion proteins can also be used to localize T cell costimulatory signals to B7H6 positive cells. K562 cells that express B7H6 were plated with CFSE-labeled human T cells and varying concentrations of a control Fc-protein (red circles), the variant NKp30-Fc protein alone (purple circles), the variant ICOSL-Fc fusion protein alone (orange circles), a wild type NKp30-ICOSL-Fc fusion protein (blue circles) or an ICOSL-NKp30-Fc fusion variant (green circles). Results shown are representative of at least two experiments, and individual points represent mean values of triplicate wells ±s.d.

To test this strategy, the engineered ICOSL domains were fused to an engineered NKp30 domain intended to provide tumor localization. NKp30 is an IgSF family member whose counter-structure is B7H6, another IgSF family member (23). The extracellular domain of NKp30 was subjected to a directed evolution campaign like the campaign conducted with ICOSL. A variant with high affinity for B7H6 was identified and fused with wild type or engineered ICOSL domains plus an effector function negative Fc domain (Figure 8A). The resultant Fc-fusion proteins (designated “ICOSL-NKp30 vIgD-Fc”) were produced in HEK-293 cells and purified by Protein A and size exclusion chromatography.

To test the function of these proteins, recombinant B7H6-Fc was coated to plates with anti-CD3 antibody and purified human T cells were added to the plates with ICOSL-NKp30 vIgD-Fc or control proteins. Effects of the proteins on proliferation and IFNγ production were assessed after 3 days in culture. Anti-CD3 plated with soluble control Fc protein, NKp30 vIgD-Fc, or ICOSL vIgD-Fc induced a similar, low level of CD4+ T cell proliferation (Figure 8B), CD8+ T cell proliferation (Figure 8C), and IFNγ production (Figure 8D). However, inclusion of ICOSL-NKp30 vIgD-Fc uniquely provided a costimulatory signal leading to increased T cell proliferation (Figures 8B,C) and IFNγ production (Figure 8D), presumably through NKp30 binding of plate-bound B7H6 tethering the ICOSL domain to the plate, analogous to what was done in experiments with plate coated ICOSL vIgD-Fc proteins (Figure 6).

To test whether this strategy could be used to localize costimulatory domains to tumor cells, K562 cells, which constitutively express B7H6 (23), were incubated with purified human T cells and a sub-optimal dose of anti-CD3 with or without ICOSL-NKp30 vIgD-Fc or control proteins. Cytokine production was evaluated after 3 days in culture. Again, ICOSL-NKp30 vIgD-Fc boosted T cell responses as determined by increase of cytokine production, whereas a fusion protein including WT ICOSL-Fc only marginally increased responses. NKp30 vIgD-Fc, ICOSL vIgD-Fc, or a control-Fc protein had no effect (Figure 8E). These data show vIgD localization domains can potentially be used to target a costimulatory vIgD to a specific cell type to enhance T cell responses.

### Delivery of Localized T Cell Costimulation via Tumor-Specific Antibody

**Figure 9:**
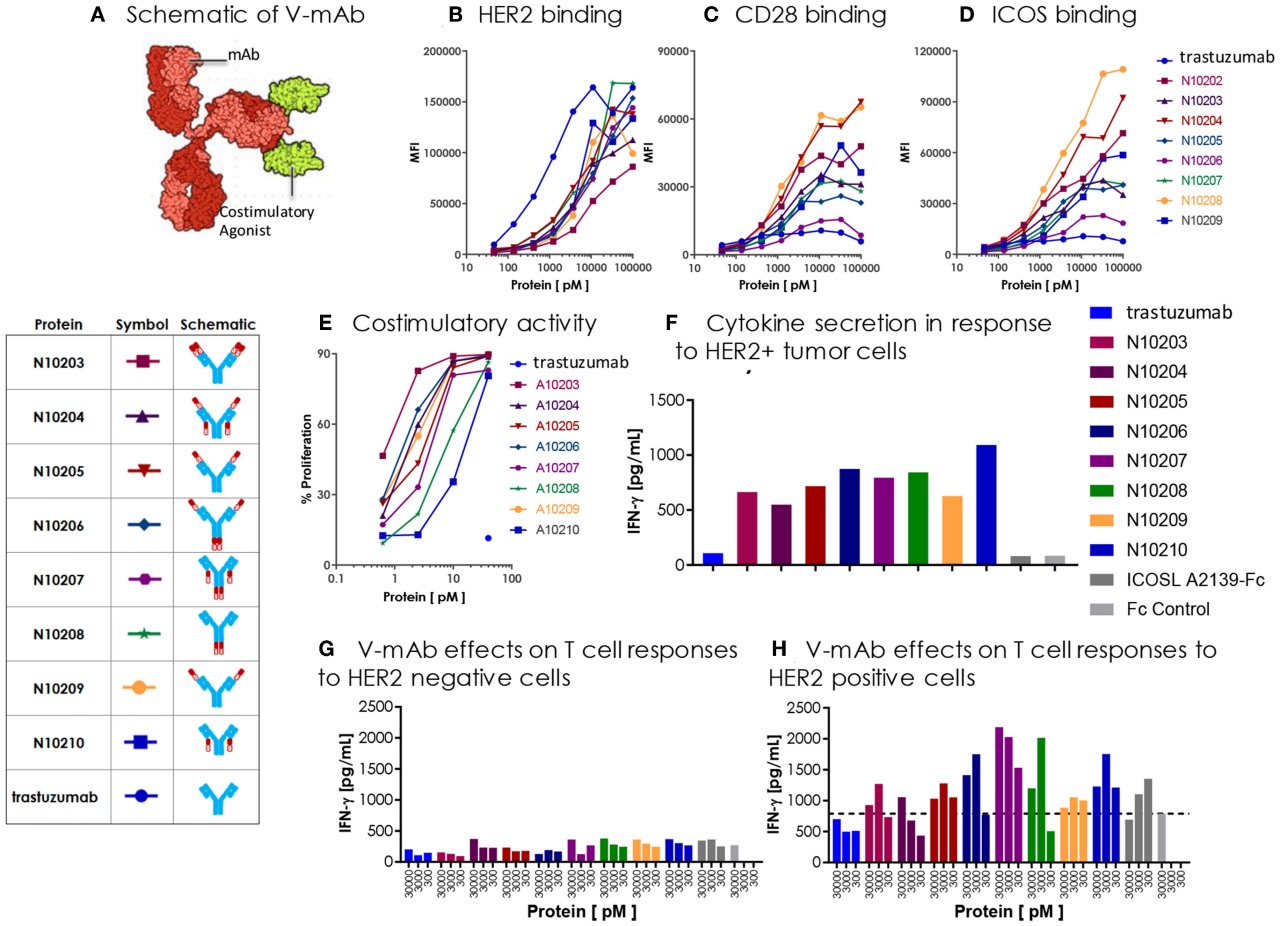
Engineered ICOSL costimulatory vIgDs can be fused to an antibody, retain binding, and provide localized costimulatory signals. **(A)** Schematic diagram of V-mAb fusion proteins. Blue structures represent heavy and light chains of the monoclonal antibody, solid red structures represent the IgV domain of an ICOSL vIgD, and open red structures are the IgC domain of an ICOSL vIgD. ICOSL-trastuzumab V-mAbs retain binding to **(B)** HER2, **(C)** CD28, and **(D)** ICOS. HEK-293 HER2 transfectants were stained with titrated amounts of indicated V-mAbs and analyzed by flow cytometry. **(E)** ICOSL-trastuzumab V-mAbs retain costimulatory activity. Titrated amounts of indicated V-mAbs were coated to plates with a fixed concentration of anti-CD3 antibody (10 nM) and incubated for 3 days with CFSE labeled T cells. Graph shows the percentage of CD8+ T cells that divided vs. protein concentrations. **(F)** ICOSL-trastuzumab V-mAbs costimulate primary human T cells in the presence of a HER2+ tumor cell line. NCI-N87 (HER2+) human gastric carcinoma cells were transduced with anti-CD3 single chain Fv (OKT3). Primary human T cells were plated with OKT3 expressing tumor cells at an E:T ratio of 1:4 and assayed 72 h later for IFN-γ production. **(G,H)** ICOSL-trastuzumab V-mAb driven T cell costimulation is dependent on HER2 expression. Primary human T cells were transduced with lentiviral vector encoding the HLA-A^*^0201 restricted TCR directed against the viral oncoprotein human papillomavirus type 16 (HPV-16) E6. Parental T2 cells **(G)** or HER2-transduced T2 cells **(H)** were pulsed with 1 ng ml^−1^ E6 peptide (29–38) for 90 min then plated with E6 TCR transduced T cells at an E:T ratio of 1:3. ICOSL-trastuzumab V-mAbs were added at the indicated concentrations and supernatants were harvested for assessment of IFNγ after 24 h. Localization of the ICOSL vIgD on HER2+ targets with the ICOSL-trastuzumab V-mAb significantly increased IFNγ production over trastuzumab alone.

As discussed above, localization of a costimulatory vIgD to tumors could have a beneficial impact on tumor attack and destruction. In addition to utilizing a tumor binding vIgD for this localization, an alternative strategy would be to fuse a costimulatory vIgD to a tumor specific antibody. Tumor specific antibodies selectively binding to tumor cells and facilitating their eradication through induction of antibody-dependent and/or complement dependent cytotoxicity (ADCC (24) or CDC (25), respectively), or targeted delivery of a toxin as antibody-drug conjugates (ADC) (26), are already widely used and there are numerous clinically-validated antibodies for this purpose. One of these clinically validated antibodies, trastuzumab (27), binds to the tumor-specific protein HER2 and was selected as a proof of concept to validate use of an antibody for providing localized costimulation through delivery of a costimulatory vIgD to tumor cells, rather than a toxin or other coupled entity. A set of proteins was generated by fusing trastuzumab with either wild type ICOSL or one of the engineered ICOSL domains. There were a limited number of combinations possible when constructing ICOSL-NKp30 vIgD-Fc and the form chosen (based on the best retention of binding activities) is shown in Figure 8A. However, there were considerably more possible combinations generating trastuzumab-ICOSL vIgD fusion monoclonal antibodies (termed “trastuzumab-ICOSL V-mAbs”) since ICOSL domains could be fused to either the N- or C-terminal ends of the heavy or light chain of the antibody. To determine the optimal configuration for trastuzumab-ICOSL V-mAbs, multiple fusion formats were explored to generate potentially active therapeutics. Trastuzumab-ICOSL V-mAbs were prepared using the A2239 engineered ICOSL domain because it was one of the more potent agonistic variants (Figures 6, 7, Table 1, and data not shown). A schematic showing the basic structure of one V-mAb and diagrams of the various specific formats tested are shown in Figure 9A.

DNA encoding each of the constructs diagrammed in Figure 9A was transfected into HEK-293 cells and secreted proteins were purified by Protein A and size exclusion chromatography (see also section Methods). The resultant trastuzumab-ICOSL V-mAbs were assessed for retention of appropriate binding properties. HEK-293 cells were transiently transfected with HER2, CD28, or ICOS expression vectors and each transfectant was then incubated with individual trastuzumab-ICOSL V-mAbs plus a secondary antibody for detection of bound reagents. HER2 binding was retained by all trastuzumab-ICOSL V-mAbs, although the magnitude of the binding was reduced somewhat (Figure 9B). Moreover, binding of trastuzumab-ICOSL V-mAbs to both CD28 (Figure 9C) and ICOS (Figure 9D) transfected cells remained largely intact, although a few forms showed some reduction in binding. Collectively, these data indicated engineered ICOSL domains could be fused to antibody heavy and/or light chains and the resultant trastuzumab-ICOSL V-mAbs largely retained counter-structure and antibody binding activity.

To confirm that ICOSL vIgD domains retained costimulatory potential when incorporated into the trastuzumab-ICOSL V-mAb format, the V-mAbs were coated to plates at varying concentrations with a sub-optimal dose of plate coated anti-CD3 antibody and CFSE-labeled T cells were added and cultured for 3 days. Figure 9E illustrates that including V-mAbs with ICOSL vIgD costimulatory domains could significantly enhance T cell proliferation compared to trastuzumab or anti-CD3 alone indicating that the ICOSL vIgD domains retained their costimulatory potential. To verify that the costimulatory capacity was dependent on the presence of HER2, NCI-N87 (HER2+) human gastric carcinoma cells were transduced with a lentivirus encoding a cell surface expressed ScFv fragment of OKT3 to generate a HER2+ cell line with the capacity to stimulate primary human T cells through their antigen receptors. These tumor cells were incubated with primary human T cells at an E:T ratio of 1:4 and varying concentrations of the various V-mAbs and IFNγ production was assessed after 3 days. Inclusion of ICOSL vIgD V-mAbs induced considerably more IFNγ production compared to native trastuzumab or a control Fc protein (Figure 9F), suggesting that ICOSL V-mAbs could provide HER2-dependent T cell costimulation. To determine whether HER2 expression was necessary for costimulation in an antigen dependent system, primary human T cells were transduced with lentiviral expression vector encoding the HLA-A^*^0201-restricted E6 TCR, which recognizes a peptide from the human papilloma virus E6 protein (28). The TAP-deficient HLA-A^*^0201+ cell line CEM.T2 was used as the antigen presenting cell in this case. Two versions of CEM.T2 cells were used: First the native cell line, which lacks HER2 expression, and second was a HER2 transduced version that expressed high levels of HER2. Inclusion of a low dose of E6 peptide with the HER2 negative native CEM.T2 cell line stimulated a low level of IFNγ production that was unaffected by inclusion of trastuzumab or any of the V-mAbs (Figure 9G). However, the HER2-expressing version of the CEM.T2 cell line triggered greatly increased levels of IFNγ when most of the V-mAbs were included but were unaffected by trastuzumab (Figure 9H). These data demonstrate that ICOSL vIgD domains fused to an anti-HER2 antibody could provide HER2-dependent costimulation of human primary T cells.

Collectively, the data demonstrate engineered ICOSL domains with enhanced ICOS/CD28 binding can deliver a costimulatory signal superior to wild type domains when immobilized on a surface. Importantly, these engineered ICOSL domains are functionally flexible enough to allow delivery of the costimulatory signal when coated to plastic or when cell bound, either through a localizing domain engineered with the vIgD platform (e.g., NKp30), or through localization mediated by binding of a monoclonal antibody (e.g., trastuzumab).

## Discussion

We have developed a directed evolution platform using IgSF proteins to generate variants with altered binding affinities and specificities, potentially creating therapeutic candidates that can be applied to a range of human disease. Optimization of wild-type IgSF protein properties to influence specific biological processes can direct selection strategies yielding an ideal, engineered protein for a specific therapeutic application. We describe a directed evolution campaign exemplifying the capabilities of our scientific platform where ICOSL was engineered to not only bind ICOS with higher affinity than the parental protein, but also to bind the non-cognate ligand CD28 with high affinity. Moreover, we demonstrate the resultant engineered ICOSL domains can be utilized in alternative molecular formats to achieve multiple therapeutic goals. Specifically, delivery of engineered ICOSL domains as a soluble ICOSL vIgD-Fc format antagonized T cell activation by inhibiting CD28 and ICOS signaling, whereas tethering of an engineered ICOSL domain to a surface or cell facilitated T cell costimulatory signaling. Importantly, we found no evidence that ICOSL vIgD domains stimulate T cells in soluble or immobilized format without antigen receptor stimulus, potentially indicating the nature of the signals provided are safely costimulatory rather than non-discriminately activating. In addition, we were unable to ascribe any function to the CTLA4 binding of the ICOSL vIgD in the assays used to characterize them. Specifically, ICOSL vIgD proteins produced identical results when *in vitro* assays were run in the presence or absence of blocking anti-CTLA4 antibodies (Supplementary Figure 3a). These observations are most likely attributable to the use of systems where the influence of CTLA4 is not significant. However, multiple methods of generating a localized, ICOSL vIgD driven, costimulatory signal were validated, including direct coating to plastic (Figure 6), transmembrane expression on the surface of a cell (Figure 7), fusion to a separate engineered localizing domain to bind plate-bound or cell surface expressed protein targets (Figure 8), and fusion of an engineered domain to a tumor specific monoclonal antibody (Figure 9).

Therapeutically, interfering with T cell costimulation has been shown to be an effective strategy for attenuating T cell responses in human autoimmune disease settings. Examples include abatacept (CTLA4-Fc) in treating rheumatoid arthritis (5), psoriatic arthritis (29) and juvenile idiopathic arthritis (30) and belatacept (an engineered version of CTLA4-Fc) in preventing transplant rejection (18, 19). However, both approved therapeutic proteins bind to CD80 and CD86 and only prevent ligand induced CD28 signaling, whereas the described ICOSL vIgD-Fc therapeutics target both CD28 and ICOS costimulatory signaling. Despite their structural similarities and engagement of common downstream signaling pathways, it has recently become clear that ICOS and CD28 play non-redundant roles in T cell activation. This has been best documented for T follicular helper (Tfh) cells, where it's been shown that CD28 plays a role in early events in Tfh differentiation, whereas ICOS is critical in maintenance of the Tfh phenotype (31). This suggests that dual antagonism of CD28 and ICOS could be particularly advantageous in autoimmune diseases where development of auto-antibodies contributes to pathogenesis since this process is particularly dependent on the contribution of Tfh cells. In this regard, we've shown that ICOSL vIgD-Fc proteins can antagonize T cells that express both CD28 and ICOS (Supplementary Figures 3b,c) as Tfh cells do.

Moreover, ICOSL vIgD-Fc antagonize costimulatory signaling better than either abatacept (Supplementary Figures 3b,c and data not shown) or belatacept (Figure 4). These *in vitro* results were validated *in vivo* using human ICOSL vIgD-Fc in a short-term immune challenge model in mice (DTH, Figure 5A) and a longer-term humanized mouse model (huPBMC-NSG GvHD; Figures 5B,C). Collectively these data suggest the soluble Fc forms of engineered ICOSL domains can be effective in controlling inflammatory diseases mediated by aberrant T cell activation.

A separate application of engineered ICOSL domains is to tether the therapeutic candidate to a cell surface to deliver a localized costimulatory signal. Tumor localized costimulation to enhance tumor-infiltrating T cell effector function is a promising therapeutic approach and targeting of both CD28 and ICOS in this way could provide therapeutic advantages as each has been shown to serve non-overlapping functions with CD28 contributing to enhanced IL2 production, cell cycle progression and survival (32, 33), while ICOS seems to play a greater role in maintaining differentiated T cell phenotypes (33). Also, most primary tumor cells lack expression of costimulatory molecules such as CD80, CD86, and ICOSL, and T cell anti-tumor responses can be compromised by lack of costimulation (34). Further, sub-optimal tumor responses to anti-PD1 monoclonal antibodies have been attributed to lack of accompanying, local costimulation (35, 36). By delivering costimulatory domains to tumor cells using a tumor-localizing vIgD (such as NKp30 localization to B7H6+ tumor cells; Figure 8) or a tumor specific monoclonal antibody (such as trastuzumab localization to HER2+ tumor cells; Figure 9), T cell responses can be enhanced in the absence of tumor-expressed costimulatory proteins. Such an approach still requires *in vivo* validation, but the remarkable specificity demonstrated *in vitro* bodes well for translation *in vivo*.

In addition to the specific example of ICOSL described here, directed evolution of numerous IgSF proteins has the potential for serially generating effective therapeutic protein candidates at a platform level. Indeed, we have used this system to develop variants of other IgSF proteins, including PD-L1, PD-L2, CD155, CD112, CD80, and others with altered specificity and affinity with multiple therapeutic applications (data not shown). Additionally, engineered domains produced by the vIgD platform have shown themselves to be “modular” in nature, allowing them to be formatted with immunoglobulin Fc, other engineered vIgD domains, and antibodies as well as expressed in engineered cellular therapeutic products. We continue to explore alternative vIgD protein engineering and formatting strategies to attain desired biological outcomes that benefit human patients in need.

## Data Availability Statement

All datasets generated for this study are included in the article/Supplementary Material.

## Ethics Statement

All animal procedures were reviewed and approved by the appropriate Institutional Animal Care and Use Committee overseeing the vivarium where the studies were conducted (Alpine Immune Sciences and The Jackson Laboratory), and followed the guidelines set forth in the 8th Edition of the Guide for the Care and Use of Laboratory Animals (National Research Council, 2011).

## Author Contributions

SL, MK, and RS prepared the manuscript. SL, LE, ER, SB, and RW performed the *in vitro* experiments. KL and SD performed and/or coordinated the *in vivo* studies. JH, SM, MW, and MR prepared and purified proteins. MK and LE performed the variant protein selections. DL prepared the structural models presented. SP supervised the work and assisted in preparation of the manuscript.

### Conflict of Interest

All authors were paid consultants (DL) or full-time employees of Alpine Immune Sciences during the course of the work presented and preparation of the manuscript.
