# Supplementary material for: Novel Immunomodulatory Proteins Generated via Directed Evolution of Variant IgSF Domains
Source: Front Immunol. 2020 Jan 21;10:3086. doi: 10.3389/fimmu.2019.03086 (PMC6985287; doi:10.3389/fimmu.2019.03086)
Supplement: Supplementary file 2 [file Table_2.docx]

**Supplementary Table 2.** Binding of human ICOSL vlgD-Fc proteins to mouse and human counter-structures by FACS-based binding assay.

| **Test Protein** | **Flow Cytometric EC50 (nM)** | | | | | | | |
| --- | --- | --- | --- | --- | --- | --- | --- | --- |
|  | **ICOS** | | **CD28** | | **CD80** | | **CD86** | |
|  | **Human** | **Mouse** | **Human** | **Mouse** | **Human** | **Mouse** | **Human** | **Mouse** |
| **WT ICOSL** | 2.6 | 4.8 | ND | 2751 | NT | NT | NT | NT |
| **A184** | 1.4 | 3 | 2 | 6.3 | NT | NT | NT | NT |
| **A2229** | 1.7 | 3.2 | 2.3 | 7 | NT | NT | NT | NT |
| **A2230** | 1.5 | 2.3 | 2.4 | 22.2 | NT | NT | NT | NT |
| **A2237** | 1.5 | 1.3 | 1.5 | 4.6 | NT | NT | NT | NT |
| **Abatacept** | NT | NT | NT | NT | 1.02 | 0.96 | 2.61 | 5.05 |
| **Belatacept** | NT | NT | NT | NT | 0.84 | 1.13 | 2.68 | 4.21 |
| NT=Not tested  ND=Not detected | | |  |  |  |  |  |  |
