# Supplementary material for: Novel Immunomodulatory Proteins Generated via Directed Evolution of Variant IgSF Domains
Source: Front Immunol. 2020 Jan 21;10:3086. doi: 10.3389/fimmu.2019.03086 (PMC6985287; doi:10.3389/fimmu.2019.03086)
Supplement: Supplementary file 3 [file Table_3.docx]

Supplementary Table 3. Monomeric protein quality as determined by percent main peak area from analytical SEC.

| Protein | % Main Peak |
| --- | --- |
| WT ICOSL - Fc | 96.6 |
| A160 | 97.7 |
| A180 | 95.5 |
| A183 | 96.8 |
| A184 | 94.5 |
| A2227 | 87.9 |
| A2229 | 95.8 |
| A2231 | 97.5 |
| A2236 | 96.4 |
| A2237 | 97.4 |
| A2239 | 97.6 |
| A2241 | 93.5 |
| A3256 | 97.1 |
| A3269 | nd^1^ |
| A3305 | 90.9 |
| A3310 | 96.0 |
| A3318 | 89.5 |
| A3321 | 95.4 |
| A3322 | 88.9 |
| WT ICOSL-NKp30 - Fc | 96.1 |
| A1174 | 94.8 |
| A1197 | 94.0 |
| A1198 | 94.9 |
| trastuzumab | 98.5 |
| A10203 | 87.9 |
| A10204 | 95.6 |
| A10205 | 95.8 |
| A10206 | 93.7 |
| A10207 | 94.3 |
| A10208 | 92.2 |
| A10209 | 90.4 |
| A10210 | 96.0 |

^1^nd, not determined
