# Supplementary figures and images for: Novel Immunomodulatory Proteins Generated via Directed Evolution of Variant IgSF Domains
Source: Front Immunol. 2020 Jan 21;10:3086. doi: 10.3389/fimmu.2019.03086 (PMC6985287; doi:10.3389/fimmu.2019.03086)

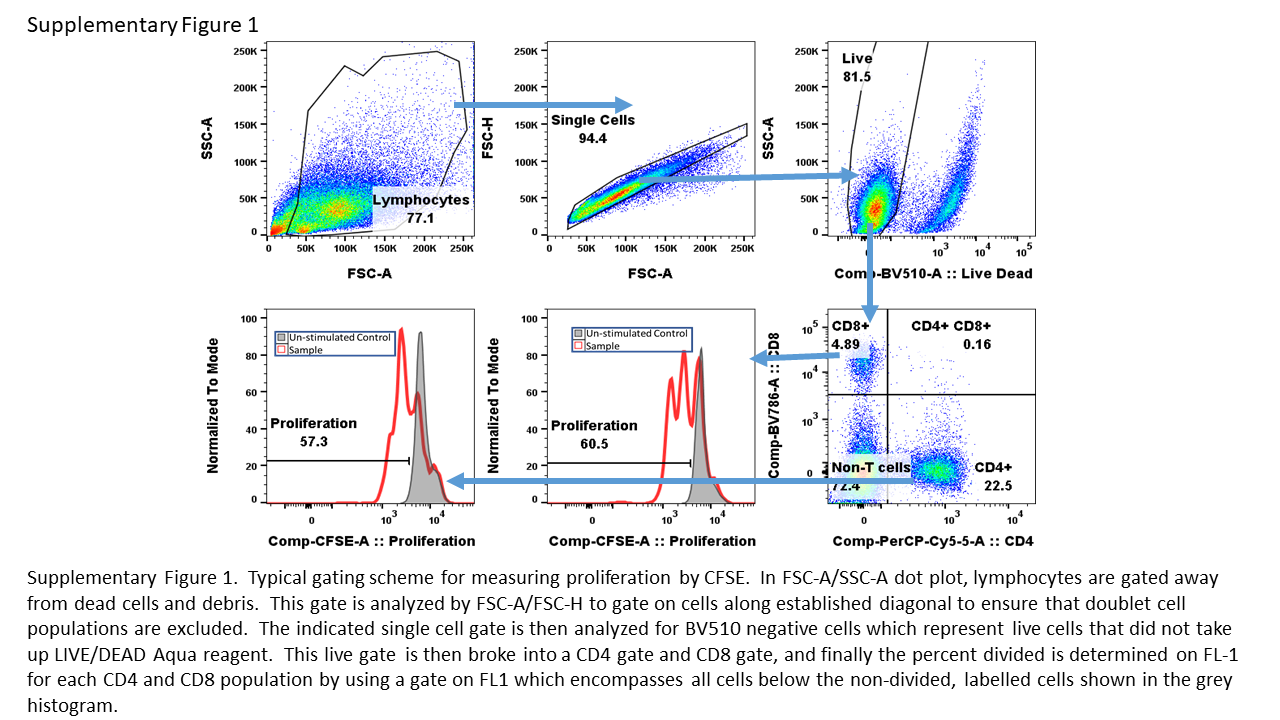

Supplement: Supplementary file 4 [file Image_1.tif]

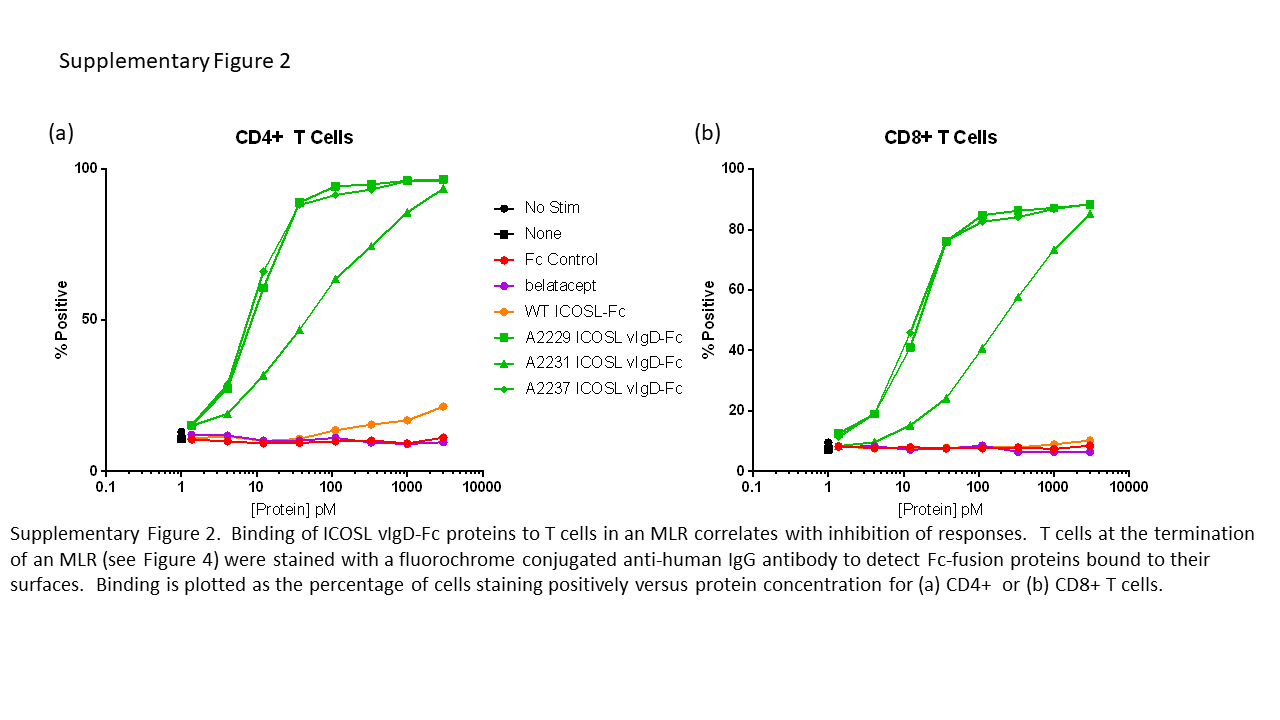

Supplement: Supplementary file 5 [file Image_2.tif]

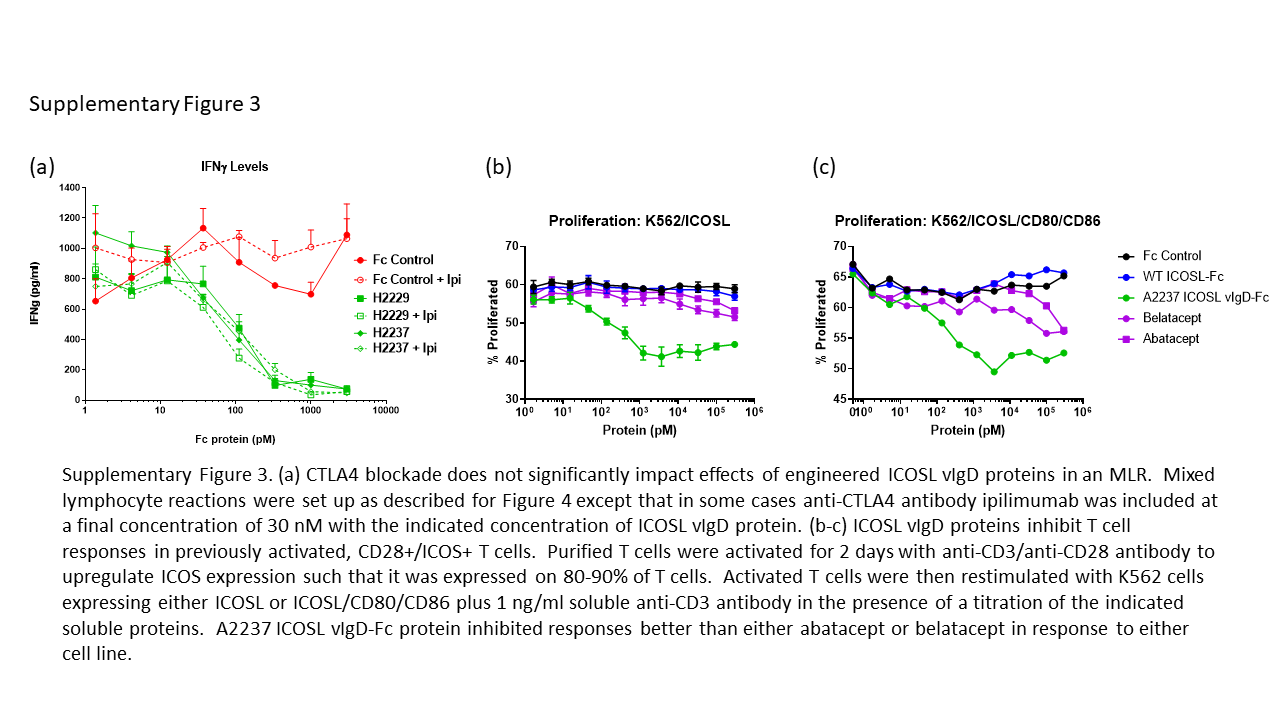

Supplement: Supplementary file 6 [file Image_3.tif]
